# Escitalopram-induced sinus bradycardia in coronary heart disease combined with depression: a case report and review of literature

**DOI:** 10.3389/fcvm.2023.1133662

**Published:** 2024-01-11

**Authors:** Liu-Cheng Li, Wen Sun, Xiao-Qin Lv, Yao-Yao Xu, Ying Hu, Jia-Na Shi

**Affiliations:** ^1^ Department of Pharmacy, Sir Run Run Shaw Hospital, Zhejiang University School of Medicine, Hangzhou, China; ^2^ Center for Clinical Pharmacy, Cancer Center, Department of Pharmacy, Zhejiang Provincial People’s Hospital, Affiliated People’s Hospital, Hangzhou Medical College, Hangzhou, China; ^3^ Department of Pharmacy, The Affiliated Hospital of Hangzhou Normal University, Hangzhou, China; ^4^ Department of Drug Monitoring and Evaluation, Zhejiang Center for Drug and Cosmetic Evaluation, Hangzhou, China; ^5^ Department of Pharmacy, The People’s Hospital of Pingyang, Wenzhou, China

**Keywords:** escitalopram, case report, sinus bradycardia, depression, coronary heart disease

## Abstract

For patients with cardiovascular disease, using the antidepressant escitalopram may lead to unexpected adverse events. Here, a rare repeated sinus bradycardia event due to escitalopram is first reported. In an 82-year-old female patient with cardiac dysfunction using digoxin, tachycardia (average heart rate of 93 beats/min) was demonstrated by electrocardiogram (ECG). She began to take escitalopram and lorazepam due to depression, but sinus bradycardia (93.7% heart rate was <60 beats/min) and sinus arrest were first detected after 3 months. Its proportion decreased to 0.1% after discontinuation of digoxin and escitalopram for 1 day, and the rhythm returned to normal 2 weeks later. After 2 months, escitalopram was prescribed again in combination with quetiapine; then, 17.1% heart rate was <60 beats/min. After escitalopram and quetiapine withdrawal, the ECG showed the heart rhythm had normalized again. No other drug changes were made during these periods. Escitalopram was deemed to be a highly possible cause of sinus bradycardia according to its Naranjo's Algorithm score. Furthermore, literature on escitalopram-mediated cardiovascular adverse events was reviewed and analyzed. Empirically, escitalopram should be discontinued immediately if iatrogenic causes cannot be ruled out. Furthermore, ECG monitoring in escitalopram-related cardiovascular adverse events is highlighted, especially in patients receiving certain drug classes simultaneously (i.e., sinoatrial node inhibitors, antipsychotics).

## 1 Introduction

Escitalopram is a well-tolerated selective serotonin reuptake inhibitor (SSRI) used to treat depressive and anxiety disorders (1). It has no inhibitory effects on cholinergic receptors, histamine receptors, or adrenergic *α* receptors, and thus, may cause few adverse drug reactions (ADR) in patients (2). The ESC working group indicated that 15%–30% of patients with coronary heart disease also have depression, and the incidence of depression in patients with coronary heart disease is 2–3 times higher than that in the general population (3). A recent meta-analysis of randomized controlled trials revealed that the probability of cardiovascular side effects associated with antidepressants could be as high as 0.89% (4). The treatment of coronary heart disease and depression may present more complicated drug-related problems (DRP) and, thus, clinical pharmacists are urgently needed to participate in the drug treatment process.

In the present case report, escitalopram-induced sinus bradycardia and sinus arrest were reported in a patient with coronary heart disease and depression. We analyzed the etiology of escitalopram-induced sinus bradycardia under the condition of drug combination and the key points of pharmaceutical care in this population. We also conducted a review of literature on escitalopram-induced ADR other than sinus bradycardia, which is not listed in the package insert of escitalopram in patients with cardiovascular disease in order to reduce and avoid DRP.

## 2 Case presentation

At the first visit to our hospital, this 82-year-old female patient said she began to develop chest tightness and shortness of breath without an obvious trigger 10 years ago. These symptoms gradually worsened due to the lack of systematic diagnosis and timely treatment. She underwent coronary angiography 5 years ago, which revealed multiple vascular stenosis and lesions, and two stents were implanted in the right coronary artery.

**Figure 1:**
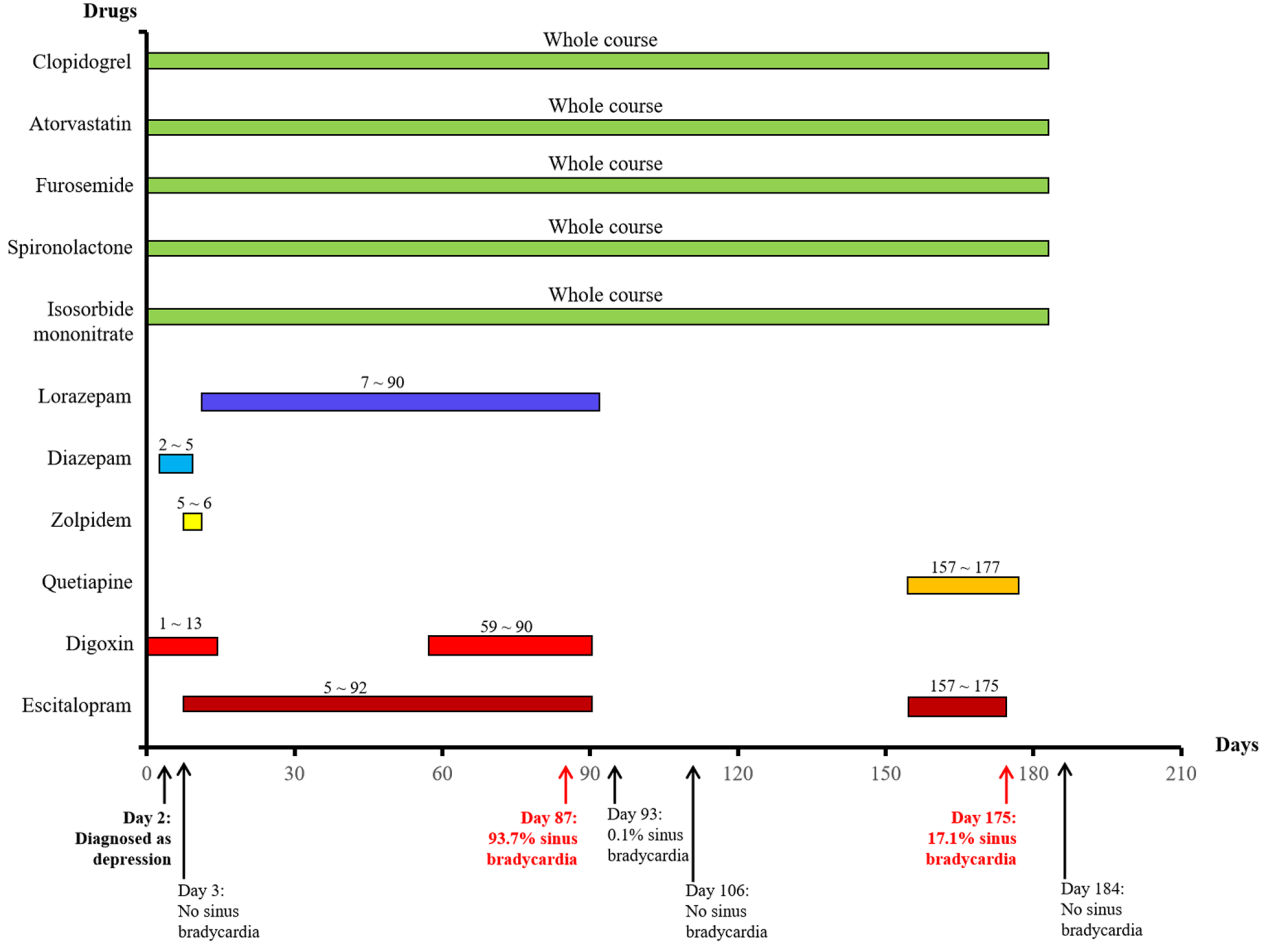
The used drugs and sinus bradycardia events by time in a patient with coronary heart disease.

**Figure 2:**
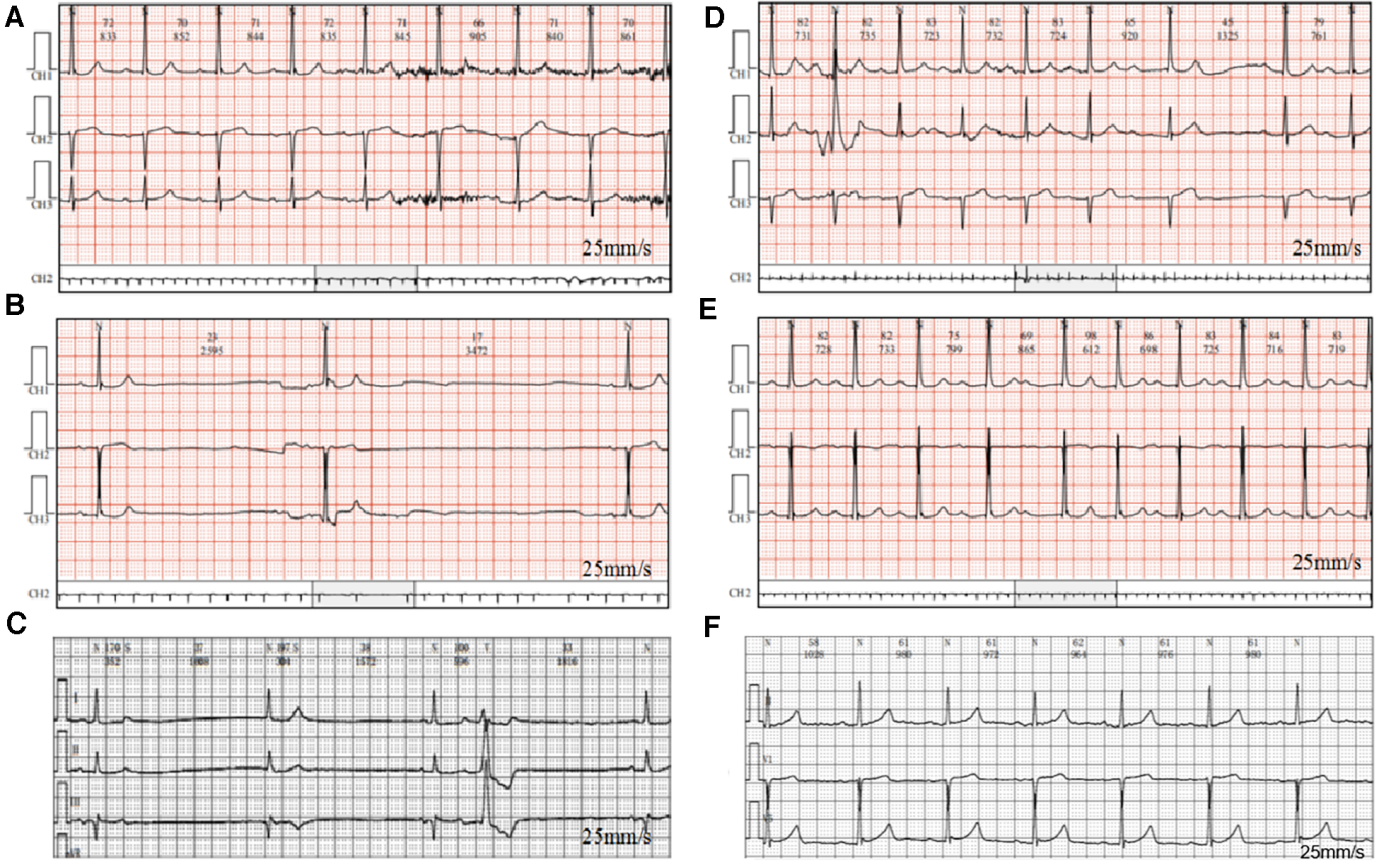
Electrocardiogram detection at different monitoring times.

She attended our hospital again 6 months ago due to chest pain and shortness of breath accompanied by nocturnal paranoia and edema of the lower extremities. The admission diagnosis included coronary atherosclerotic heart disease, status after coronary stent implantation, and NYHA III cardiac function. Coronary artery calcium was detected by electron-beam computed tomography. Atherosclerotic plaques in the proximal left anterior descending branch and mixed plaques in the proximal left circumflex branch showed mild to moderate lumen stenosis. Meanwhile, the liver enzyme levels that reflected liver function and the creatinine level that reflected renal function were examined. We found that the levels of glutamic-pyruvic transaminase (17 U/L), glutamic oxalacetic transaminase (20 U/L), glutamyl transpeptidase (21 U/L), alkaline phosphatase (92 U/L), total bilirubin (10 μmol/L), and creatinine (98.5 μmol/L) were all within the normal range in our hospital. However, the patient did not have an echocardiogram examined or cardiac markers tested. The 24-h Holter ECG monitoring of the patient revealed sinus rhythm accompanied by ST segment changes and atrial tachycardia but without bradycardia or sinus pause/arrest. Then, the patient was treated with symptomatic supportive treatments including clopidogrel, atorvastatin, furosemide, spironolactone, and isosorbide mononitrate. Due to the symptoms of cardiac dysfunction (difficulty breathing, limited physical activity, and fluid retention) and atrial tachycardia, digoxin was also prescribed on day 1. All the drugs used for treating the patient are shown in Figure 1. She developed symptoms of insomnia and was first diagnosed with depression; she began diazepam treatment on day 2. The next day, electrocardiogram (ECG) detection showed tachycardia and second-degree atrioventricular block type I with an average heart rate of 93 beats/min, but with no sinus bradycardia event (Figure 2A). On day 5, diazepam was replaced by zolpidem, while escitalopram (5 mg once a day) was first prescribed in the treatment due to unimproved symptoms (sleep disturbances and anxiety). On day 7, zolpidem was replaced by lorazepam, and the dose of escitalopram was increased to 10 mg once a day. On day 14, the dose of escitalopram was increased to 15 mg once a day until day 92. Laboratory test results were normal, including potassium and magnesium serum levels. A detailed description of the use of drugs during the onset of sinus bradycardia is shown in Table 1.

**Table 1:** The usages and manufacturers of drugs used.

Drugs	Dosage (mg)	Frequency	Manufacturer
Clopidogrel	75	qd	Sanofi Pharmaceutical Co., Ltd., France
Atorvastatin	20	qd	Pfizer Pharmaceuticals Ltd., USA
Furosemide	20	qd	Shanghai Zhaohui Pharmaceutical Co., Ltd., China
Spironolactone	20	qd	Hangzhou Minsheng Pharmaceutical Co., Ltd., China
Isosorbide mononitrate	20	bid	Shandong Lunan Better Pharmaceutical Co., Ltd., China
Digoxin	0.125	qd	Shanghai Xinyi Pharmaceutical Co., Ltd., China
Diazepam	2.5	qn	Shandong Xinyi Pharmaceutical Co., Ltd., China
Escitalopram	5	qd	H. Lundbeck A/S, Denmark
Zolpidem	5	qn	Sanofi Pharmaceutical Co., Ltd., France
Escitalopram	10	qd	H. Lundbeck A/S, Denmark
Escitalopram	15	qd	H. Lundbeck A/S, Denmark
Lorazepam	0.5	qn	Atlantic Pharmaceutical Co., Ltd., Thailand
Digoxin	0.125	qd	Shanghai Xinyi Pharmaceutical Co., Ltd., China
Escitalopram	11.25	qd	H. Lundbeck A/S, Denmark
Quetiapine	25	qd	AstraZeneca Pharmaceutical Co., Ltd., UK
Escitalopram	10	qd	H. Lundbeck A/S, Denmark

qd, once a day; bid, twice a day; qn, once a night.

On day 87, the patient developed a substantial proportion of bradycardia below the focused level (heart rate <60 beats/min) and sinus arrest, with second-degree type II sinus atrial block with an average heart rate of 53 beats/min by the second ECG detection (Figure 2B). The duration of sinus bradycardia (heart rate <60 beats/min) was 93.7% of the total time. The slowest heart rate was 32 beats/min, while the fastest heart rate was 69 beats/min. However, the ECG report revealed similar percentages of low heart rate in the daytime and nighttime (sleep) periods. On day 90, lorazepam was suspended but the use of escitalopram was continued. On day 92, escitalopram was also suspended. The next day, we rechecked the ECG and it showed continued sinus arrest with first-degree atrioventricular block with an average heart rate of 67 beats/min (Figure 2C). Surprisingly, sinus bradycardia significantly decreased from 93.7% (before escitalopram discontinuation) to 0.1% (after escitalopram discontinuation). The doctors concluded that escitalopram might have been the cause of the sinus bradycardia events, and then escitalopram was discontinued. On day 106, sinus bradycardia disappeared but the atrioventricular conduction remained, showing prolonged PR and second-degree Mobitz type I blockage (Figure 2D). Furthermore, we found that the levels of glutamic-pyruvic transaminase (8 U/L), glutamic oxalacetic transaminase (13 U/L), glutamyl transpeptidase (14 U/L), alkaline phosphatase (99 U/L), total bilirubin (8.1 μmol/L), and creatinine (103.8 μmol/L) were all within the normal range in our hospital.

On day 157, the patient felt anxiety and subsequently purchased escitalopram (11.25 mg every day) and quetiapine to treat anxiety outside of our hospital. Unfortunately, the patient said she experienced chest distress and shortness of breath again with worse symptoms, accompanied by fatigue and conscious numbness of the limbs on day 173. The dynamic ECG in the local hospital showed sinus rhythm, paroxysmal atrial fibrillation, occasional R-R long interval, paired and short atrial tachycardia in atrial premature beats, and dual ventricular premature beats. After symptomatic treatment, she showed no significant improvement and was admitted to our hospital again. During this hospitalization, escitalopram and quetiapine were continued, but the dose of escitalopram was decreased to 10 mg on day 175 and then discontinued, while quetiapine was stopped on the third day. On day 175, the duration of sinus bradycardia was 17.1% of the total monitoring times, accompanied by intermittent first-degree atrioventricular block by dynamic ECG monitoring (Figure 2E). The slowest heart rate was 52 beats/min, while the fastest heart rate was 98 beats/min, with an average heart rate of 74 beats/min according to ECG detection. Meanwhile, the ECG report revealed that the bradycardia events mainly occurred from 22:00 to 05:00. One week after escitalopram discontinuation, the last ECG showed the sinus rhythm with an average heart rate of 60 beats/min and first-degree atrioventricular block (Figure 2F). Furthermore, we detected that the levels of glutamic-pyruvic transaminase (13 U/L), glutamic oxalacetic transaminase (17 U/L), glutamyl transpeptidase (14 U/L), alkaline phosphatase (124 U/L), total bilirubin (11 μmol/L), and creatinine (76.4 μmol/L) were all within the normal range in our hospital. The levels of cardiac troponin I (0.007 μg/L) and B natriuretic peptide (45.1 pg/ml) were also in the normal range. The patient obtained a Naranjo's Algorithm ADR probability score of 7, which is used to assess whether there is a causal relationship between an adverse drug experience and a drug, using a simple questionnaire to assign probability scores (5). If the total Naranjo's Algorithm score lies between 5 and 8, the causal relationship is “highly possible”; if the total score is greater than or equal to 9, the causal relationship is “definite”. Thus, escitalopram use was deemed to be a “highly possible” cause of the patient's sinus bradycardia based on the Naranjo's Algorithm ADR probability score.

## 3 Discussion

Cardiovascular disease is one of the leading causes of death worldwide (6, 7). Depression has been shown to have a causal relationship with the occurrence and prognosis of cardiovascular disease (8–10). Furthermore, depression after cardiovascular disease has been reported to be associated with an increased risk of adverse events (11, 12). Compared with traditional treatments, Psycho-Cardiology therapy improved depression symptoms and long-term outcomes in patients with coronary heart disease (13). It suggests the importance of synergistic treatment of depression and cardiovascular diseases.

Escitalopram is widely used in primary care for a variety of psychiatric disorders, such as depression and anxiety (14). It was well tolerated in clinical practice, but it also led to various adverse effects, including the common neurological, psychopathic, and gastrointestinal ADR, as well as the recent reports on escitalopram-derived hepatitis and epistaxis, which were rarely mentioned (14, 15). Reporting ADR can inform the rational use of escitalopram and further improve patient drug management. In this study, we reported a rare case of repeated sinus bradycardia due to escitalopram in combination with other drugs.

The patient experienced the first sinus bradycardia event, sinus arrest, after raising the escitalopram dose from 5 mg to 15 mg per day, and returned to normal after the interruption of escitalopram. In the beginning, the patient was prescribed digoxin because of cardiac dysfunction. A sinus rhythm of 93 beats/min was demonstrated by the first dynamic ECG detection but with no sinus bradycardia evidence 2 days later. The patient then began to take escitalopram accompanied by lorazepam due to her new depression diagnosis, but sinus bradycardia (93.7% of heart rate was <60 beats/min) was detected by the second ECG 3 months later. Surprisingly, the proportion of sinus bradycardia significantly decreased to 0.1% after escitalopram discontinuation for 1 day and the rhythm returned to normal with no sinus bradycardia 2 weeks later. After 2 months, escitalopram was prescribed again in combination with quetiapine, and then another sinus bradycardia event occurred. However, the bradycardia disappeared 1 week after escitalopram withdrawal. Scoring according to the Naranjo Algorithm probability scale revealed a highly probable relationship between escitalopram and sinus bradycardia.

DRP including drug-induced ADR interferes with expected clinical treatment outcomes and should also be an important risk management factor to increase patient safety (16, 17). Multiple studies have been devoted to evaluating the harmful effects of antipsychotic drugs during the treatment of nervous disorders (18, 19). From the pharmacovigilance system of EudraVigilance, 8% of individual ADR reports were escitalopram, while the proportion was 13% from the Food and Drug Administration Adverse Events Reporting System (20). In a retrospective analysis of a tertiary hospital, escitalopram (6.1%) had the highest incidence of adverse reactions among antidepressants (21). Further evidence indicated that escitalopram was grouped as CYP2C19 substrate, which is associated with ADR related to modulating the autonomic nervous system, seizure, and pain (22). Thus, differences in the CYP2C19 genotype can affect the serum concentration of escitalopram, thus affecting the ADR. This may be one reason for escitalopram-induced adverse cardiac reactions, but the CYP2C19 genotype was not analyzed in the patient in this study. Furthermore, the arrhythmia caused by escitalopram may be attributed to its cardiotoxic metabolite S-didesmethylcitalopram (23).

In this case, when the patient received escitalopram accompanied by digoxin and lorazepam for 1 month, the patient developed sinus bradycardia. Prior to this, there was no evidence of sinus bradycardia. Costa and colleagues showed that the oral intake of lorazepam did not affect heart rates (24). Digoxin had a narrow therapeutic margin and potentially led to severe cardiovascular adverse effects. Other drugs that lengthen the QT interval or slow cardiac conduction may induce the cardiac adverse effects of digoxin (25). However, the abnormality of ECG described obvious alleviation when both digoxin and escitalopram were stopped for this patient on day 90. To our surprise, sinus bradycardia disappeared when escitalopram and digoxin were discontinued for 2 weeks, suggesting that escitalopram and digoxin may have induced the ADR. Later, the sinus bradycardia event was rediscovered when escitalopram was combined with quetiapine to treat depression but disappeared again when escitalopram and quetiapine were suspended. From this perspective, we inferred the conclusion that the cause of sinus bradycardia in this patient might have been the combined effect of escitalopram and other drugs that may cause bradycardia or antipsychotic drugs. Until now, emerging data on case series on escitalopram-induced typical ADR in patients with depression and cardiovascular disease have been continuously reported (Table 2). In addition to bradycardia during escitalopram use, the first-degree atrioventricular block and second-degree type I atrioventricular block were also detected by electrocardiogram in this case. *In vivo* study has demonstrated that escitalopram treatment could cause a decrease in heart rate, which manifests as a significant decrease in sympathetic components and a significant increase in parasympathetic components of the autonomic nervous imbalance (38). This may be a possible pathogenesis of escitalopram-related bradycardia. Furthermore, we found that higher therapeutic doses of escitalopram (15 mg, qd) led to more serious sinus bradycardia (93.7% sinus bradycardia) than escitalopram (11.25 mg, qd), which was associated with 17.1% sinus bradycardia. This indicates that lowering therapeutic doses may help to prevent adverse events.

**Table 2:** Case reports of escitalopram-induced ADR in patients experienced cardiovascular disease.

Articles	Gender	Age	Disease history/diagnosis	Suspected drugs (usage)	Potassium level	Magnesium level	ADR
Kumar et al. (26)	Female	54	Mitral valve prolapse, cardiopulmonary arrest, depression	Escitalopram (Not provided)	Normal	Normal	Torsade de Pointes and cardiac arrest
Fischer et al. (27)	Male	22	Aortic stenosis, diastolic heart failure, and depression	Escitalopram (10 mg, qd)	Not reported	Not reported	Episodes of arm and leg tremors with associated stiffness and difficulty speaking
Pressman et al. (28)	Female	56	Grade I meningioma, depression	Escitalopram (20 mg, qd) and estrogen (Not provided)	Not reported	Not reported	Spontaneous intracranial hemorrhage
Yamagishi et al. (29)	Female	73	Hypertension, anxiety disorder	Escitalopram (Not provided) and lorazepam (Not provided)	Normal	Not reported	Depleted afterload, increased gradient of left ventricular outflow tract obstruction, and cardiac arrest
Farkas et al. (30)	Female	25	Tachycardia, anxiety, and depression	Escitalopram (serum level of 290 ng/ml) and lamotrigine (serum level of 59.3 μg/ml)	Not reported	Not reported	Refractory QRS prolongation and left bundle-branch block
Diken et al. (31)	Male	59	Acute anterior myocardial infarction, chronic obstructive pulmonary disease, diabetes mellitus, and depression	Escitalopram (10 mg, qd) and Aldactazide (50 mg, qd)	Normal	Not reported	Hyponatremia
Singh et al. (32)	Male	90	Hypertension, cerebrovascular accidents, dementia, and stage 3 chronic kidney disease	Escitalopram (10 mg, qd), aspirin (81 mg, qd), clopidogrel (75 mg, qod), quetiapine (25 mg, qd), and memantine (10 mg, qd)	Hypokalemia	Not reported	High-grade atrioventricular block
Martini et al. (33)	Male	27	Sinus tachycardia, depression, anxiety, and post-traumatic stress disorder	Escitalopram (30 mg, qd) and metaxalone (800 mg, qid)	Not reported	Not reported	Ocular clonus, inducibl clonus, agitation, and diaphoresis
Schreffler et al. (23)	Female	16	Incomplete right bundle branch block and depression	Escitalopram (500 mg, once), tramadol/acetaminophen (Not provided), and hydrocodone/acetaminophen (Not provided)	Normal	Normal	QRS complex widening and QT interval prolongation
Tseng et al. (34)	Female	42	manic episode, and depression	Escitalopram (5 mg, qd)	Not reported	Not reported	QT prolongation
Baranchuk et al. (35)	Male	52	Hypertension, asthma, reflux disease, and depression	Escitalopram (140 mg, qd), morphine (30 mg, qd), and zopiclone (15 mg, qd)	Hyperkalemia	Normal	Prolonged corrected QT interval
Page et al. (36)	Female	34	Acute decompensated heart failure and depression	Escitalopram (10 mg, qn)	Hypokalemia	Not reported	Restless legs syndrome
Kurne et al. (37)	Male	53	Venous thromboembolism, anxiety, and depression with psychotic features	Escitalopram (20 mg, qd) and alprazolam (Not provided)	Not reported	Not reported	Popliteal venous thrombosis
Our case	Female	82	Coronary heart disease and depression	Escitalopram (15 mg or 11.25 mg, qd), lorazepam (0.5 mg, qn), and quetiapine (25 mg, qd)	Normal	Normal	Sinus bradycardia and sinus arrest

Citalopram consists of two stereostructures, one stereoisomer is S-Citalopram (escitalopram) and the other isomer is R-Citalopram (no drug activity), each of which is 50%. The cross-sectional registry study found that the prevalence of QT-prolonging drugs included citalopram and escitalopram, which represent 14.5% and 3.9% of the study population, respectively (39), confirming that escitalopram had fewer adverse reactions than citalopram. However, a recent systematic review and meta-analysis involving 1,141 patients (573 experimental and 568 control) in randomized controlled trials showed that citalopram has positive effects on the left ventricular ejection fraction and N-terminal pro-B-type natriuretic peptide in patients with depression combined with chronic heart failure; meanwhile, no obvious adverse drug reactions were observed (40). It highlights the importance of real-world case studies as an addition to randomized controlled trials in the discovery of ADR.

The metabolism of escitalopram is mainly mediated by cytochrome CYP 2C19 in the liver. Thus, liver function impairment would reduce the metabolic clearance of escitalopram, which may have greater treatment effects, but the adverse effects are also greater. Oral digoxin is excreted through the kidney, and the blood concentration of digoxin increases with the decrease of the glomerular filtration rate. Therefore, decreased renal function can increase the blood concentration of digoxin and increase the risk of drug poisoning. Both liver and kidney function were within normal range before the initial use of escitalopram and after the suspension of escitalopram. Though we did not follow up on these tests on the day of bradycardia, we could infer from the available data that the liver and kidney function should be normal during escitalopram use.

Until now, there have been no official notices or expert guidelines regarding ECG monitoring in patients taking SSRIs like escitalopram, probably due to the relative rarity of severe arrhythmias. In an early study in 2000, the first case of sinus bradycardia was caused by pure citalopram overdose (800 mg), lasting up to 6 days with severe hypotension and intermittent syncope (41). The blood level of escitalopram during the bradycardia process would definitively prove whether the dose of escitalopram is too high in this case. However, the blood concentration of escitalopram was not investigated due to the lack of its availability. A baseline ECG is usually done before the initiation of antidepressant therapy with SSRI.

However, there may be other possible causes of bradyarrhythmia that cannot be excluded. Firstly, we do not know if the patient has sick sinus syndrome (SSS) or pre-existing sinus node dysfunction, which may lead to possible intermittent episodes. Secondly, the degenerative process of dual locations of the conducting system (sinus node and atrioventricular node) in elderly patients may also be the reason, as the ECGs in this case implied that there were dual conduction abnormalities located in the sinus node and atrioventricular node. Escitalopram may only attack a single site. Thirdly, the bradycardia disappeared after escitalopram and digoxin were discontinued during the first bradycardia attack. Previously, 93.7% of sinus bradycardia was observed, but the effect of digoxin cannot be ruled out because we did not monitor the blood concentration of digoxin. The second bradycardia occurred when the patient was not taking digoxin, but only 17.1% of sinus bradycardia was present. Eliminating the influence of detection timing, this may suggest that the combination of escitalopram with other drugs, such as digoxin, may counteract the additive effect of escitalopram-induced bradycardia. Fourthly, the patient had coronary heart disease, and bradycardia could have been due to sinoatrial node ischemia. In addition, the dynamic imbalance of myocardial demand and coronary blood supply may also lead to intermittent ischemia. Nevertheless, the records of angina symptoms and cardiac biomarkers (troponin level) were lacking and only one test was reported on the level of cardiac troponin I, which was a normal value. Last but not least, during these two bradycardia events, we did not test the patient's thyroid function in time to rule out the possibility of bradycardia associated with hypothyroidism. This is the shortcoming and limitation of real-world research like this study.

Although there are many possibilities that may affect bradycardia, physicians should remain aware of cardiac ADR in patients with cardiovascular diseases, and clinical pharmacists should provide full-process pharmaceutical care for screening and timely treatment of cardiovascular adverse events. Pharmacokinetic interactions between over-the-counter drugs and antidepressants including escitalopram may further increase the severity of side effects of the latter, which, clinically, is worth highlighting (42). It is also recommended that early and close ECG surveillance is necessary for early identification and treatment of arrhythmias induced by escitalopram.

## 4 Conclusion

To the best of our knowledge, this is a rare report of a repeated sinus bradycardia event induced by escitalopram in combination with other drugs. The possibility of pharmacodynamic interactions between escitalopram and the particular drug classes (i.e., sinoatrial node inhibitors, antipsychotics) may pose a higher risk of cardiac adverse effects. Therefore, patients receiving these drug combinations should be provided with surveillance measures, including routine ECG monitoring to screen for any possible cardiovascular adverse events to facilitate early detection.

## Data Availability

The original contributions presented in the study are included in the article/Supplementary Material, further inquiries can be directed to the corresponding author.
